# A hypoxia- and lactate metabolism-related gene signature to predict prognosis of sepsis: discovery and validation in independent cohorts

**DOI:** 10.1186/s40001-023-01307-z

**Published:** 2023-09-04

**Authors:** Yaojun Peng, Qiyan Wu, Xinhuan Ding, Lingxiong Wang, Hanpu Gong, Cong Feng, Tianyi Liu, Haiyan Zhu

**Affiliations:** 1 Medical School of Chinese PLA General Hospital, Beijing, China; 2 Department of Emergency, The First Medical Center, Chinese PLA General Hospital, 28th Fuxing Road, Beijing, China; 3 Institute of Oncology, The Fifth Medical Centre, Chinese PLA General Hospital, Beijing, China

**Keywords:** Sepsis, Prognosis, Hypoxia, Lactate metabolism, Transcriptomic analysis

## Abstract

**Background:**

High throughput gene expression profiling is a valuable tool in providing insight into the molecular mechanism of human diseases. Hypoxia- and lactate metabolism-related genes (HLMRGs) are fundamentally dysregulated in sepsis and have great predictive potential. Therefore, we attempted to build an HLMRG signature to predict the prognosis of patients with sepsis.

**Methods:**

Three publicly available transcriptomic profiles of peripheral blood mononuclear cells from patients with sepsis (GSE65682, E-MTAB-4421 and E-MTAB-4451, total *n* = 850) were included in this study. An HLMRG signature was created by employing Cox regression and least absolute shrinkage and selection operator estimation. The CIBERSORT method was used to analyze the abundances of 22 immune cell subtypes based on transcriptomic data. Metascape was used to investigate pathways related to the HLMRG signature.

**Results:**

We developed a prognostic signature based on five HLMRGs (*ERO1L*, *SIAH2*, *TGFA*, *TGFBI*, and *THBS1*). This classifier successfully discriminated patients with disparate 28-day mortality in the discovery cohort (GSE65682, *n* = 479), and consistent results were observed in the validation cohort (E-MTAB-4421 plus E-MTAB-4451, *n* = 371). Estimation of immune infiltration revealed significant associations between the risk score and a subset of immune cells. Enrichment analysis revealed that pathways related to antimicrobial immune responses, leukocyte activation, and cell adhesion and migration were significantly associated with the HLMRG signature.

**Conclusions:**

Identification of a prognostic signature suggests the critical role of hypoxia and lactate metabolism in the pathophysiology of sepsis. The HLMRG signature can be used as an efficient tool for the risk stratification of patients with sepsis.

**Supplementary Information:**

The online version contains supplementary material available at 10.1186/s40001-023-01307-z.

## Introduction

Sepsis is defined as a life-threatening organ dysfunction caused by a dysregulated host response to infection [1]. It is estimated as the leading cause of death in critically ill patients, and mortality significantly increases when organ failure occurs [2]. The functions of essential organs, including lung, kidney, liver, and heart, are often interdependent. This interdependence is particularly evident in cardiovascular failure, which reduces systemic blood circulation and results in tissue hypoxia and metabolic imbalance [3]. Lactate is traditionally interpreted as a critical player in energy use [4], and is thus a marker of tissue hypoxia. In clinical settings, the measurement of serum lactate levels is a routine test during the management of critical illnesses, such as sepsis and septic shock [5]. Serum lactate levels have valuable usage in evaluating disease severity, estimating treatment response, and predicting prognosis [5]. It has been widely accepted that there is a strong and positive correlation between lactate levels and disease severity, morbidity, and mortality in sepsis [6]. Past research has shown that elevated lactate levels were significantly associated with increased mortality rate in sepsis, whereas reduced lactate levels after treatment could be a predictor of reformed clinical outcomes [7]. However, given the complexity of lactate metabolism and clearance in the pathophysiological conditions of human body, especially in sepsis, the clinical use of lactate is not as simple as recommended by some guidelines [8]. It has been traditionally accepted that elevated serum lactate concentrations in sepsis is derived from anaerobic metabolism in the conditions of tissue malperfusion and hypoxia. However, accumulating evidence suggests that anaerobic metabolism may not be the primary or exclusive source of lactate production [9]. On one hand, enhanced adrenergic stress can cause accelerated aerobic glycolysis resulting in a significant elevated lactate in septic patients [10, 11]. On the other hand, cytopathic hypoxia and mitochondrial impairment have been recognized as additional causes, although the exact mechanism remains unexplored [12, 13]. Therefore, further understanding of the molecular consequences of hypoxia and the pathophysiological processes of lactate metabolism and clearance in sepsis would help discover effective targets for therapeutic intervention.

Over the past decade, omics technologies based on genomic, transcriptomic, proteomic and metabolic profiling have shown great promise in providing insights into the molecular mechanisms of sepsis [14, 15]. Several investigators have successfully subgroup patients with sepsis based on biological similarities defined by transcriptomic profiling of peripheral blood mononuclear cells (PBMCs) using discovery-based approaches [16, 17]. Moreover, transcriptome analysis of PBMCs in sepsis research has revealed that hypoxia- and lactate metabolism-related genes (HLMRGs) are fundamentally dysregulated in sepsis, which are of great predicting potential [18–21].

In the current study, we identified differentially expressed HLMRGs by analyzing the transcriptomic data of PBMCs from patients with sepsis, based on which we developed and validated a gene model for predicting prognosis of patients with sepsis. The correlation of the predicting model with immune cell infiltration and the related signaling pathways were also investigated to explore the underlying biological mechanisms. This gene expression model is reflective of the individual’s underlying biological response and immune status.

## Methods

### Data collection and preprocessing

Gene expression profiles (GSE65682, E-MTAB-4421, and E-MTAB-4451) of PBMCs from sepsis patients and the corresponding clinical information are retrieved from the GEO (https://www.ncbi.nlm.nih.gov/geo/) and ArrayExpress (https://www.ebi.ac.uk/biostudies/arrayexpress) databases. In GSE65682, blood samples were collected within 24 h of admission to critical care, and quantification of gene expression was performed using the Affymetrix Human Genome U133 Plus 2.0 Array [22]. A total of 42 healthy controls and 479 sepsis patients with available 28-day follow-up information from GSE65682 were acquired. Both E-MTAB-4421 and E-MTAB-4451 were obtained from the GAinS study [23]. These two cohorts used the same inclusion or exclusion criteria, and gene expression was quantified using the same microarray type (GPL10558 Illumina HumanHT-12 V4.0), so they were combined. A total of 371 sepsis patients with available 28-day follow-up data from the combined dataset were acquired. The baseline clinical information of patients included in this study is presented in Additional file 1: Table S1.

To preprocess of the gene expression data, we first transformed the probes into gene symbols according to the annotation file provided by the platform manufacturer. We remove those probes without corresponding gene symbols. We averaged the values of multiple probes corresponding to a same gene as the actual intensity of this gene. Next, we merged GSE65682, E-MTAB-4421, and E-MTAB-4451 to adjust batch effects using the Combat method [24], which was executed by R software (version 4.1.2).

### Candidate gene selection

The selection of HLMRGs was based on a previously published study [25], where a set of HLMRGs were collected by searching the Molecular Signatures Database [26] (https://www.gsea-msigdb.org/gsea/msigdb/index.jsp) for relevant gene collections using “hypoxia” and “lactic” as keywords. A total of 764 HLMRGs were acquired after filtering, combining gene sets, and deleting duplicate genes. A full list of these HLMRGs is summarized in Additional file 2: Table S2.

### Development and validation of a prognostic HLMRG signature

GSE65682 was designated as the discovery set, and the combined dataset of E-MTAB-4421 and E-MTAB-4451 was assigned as the validation set. HLMRGs differentially expressed between healthy controls (*n* = 42) and patients with sepsis (*n* = 479) in the discovery set were screened using the *limma* R package. The cutoff value was set as | log_2_ (fold change) |≥ 1 and adjusted *P* < 0.05.

Cox regression (univariate and multivariate) and LASSO estimation were used to filter significant prognostic genes from the identified HLMRGs, as previously described [27, 28]. First, we performed univariate Cox regression analysis to screen HLMRGs of prognostic value, with a screening threshold of *P* < 0.05. Next, LASSO estimation was used to simultaneously achieve variable shrinkage using the *glmnet* R package [29]. The optimal values of the penalty parameter lambda in the LASSO estimation were obtained through ten-times cross-validations [30]. Finally, we obtained a best-fitting prognostic model by subsequently performing multivariate Cox regression analysis using the *survminer* R package.

Each patient’s risk score was calculated as the genes’ coefficients in the multivariate Cox regression multiplied by their expression levels. Patients were categorized into high- and low-risk groups based on the median risk score of the discovery set. The 28-day survival curves of the high- and low-risk groups were drawn based on the Kaplan–Meier estimate, and the survival difference was compared by the log-rank test. We also performed receiver operating characteristic (ROC) analysis to evaluate the sensitivity and specificity of the survival prediction using the *survivalROC* R package.

### Estimation of immune cell subtypes

The CIBERSORT method is a widely used bioinformatics tool for estimation of immune cell abundances from gene expression profiles [31]. We assessed immune cell compositions of 22 subtypes by applying the CIBERSORT algorithm to gene expression profiles, which is executed using the *IOBR* R package [32].

### Pathway enrichment analysis

To investigate the signaling pathways associated with the HLMRG signature, differentially expressed genes (DEGs; screening criteria: | log_2_ [fold change] |≥ 1 and adjusted *P* < 0.05) between the high- and low-risk groups were identified. The gene ontology (GO) analysis was performed based on the identified DEGs using Metascape [33], a wildly-accepted online tool for functional enrichment (http://metascape.org). For a given gene list, pathway and process enrichment analyses were carried out using three ontology sources: Biological Processes, Cellular Components, and Molecular Functions. All the genes in the genome were used as the enrichment background. Terms with a *P*-value less than 0.01, a minimum count of three, and an enrichment factor over 1.5 were collected and grouped into clusters based on their membership similarities. The enrichment factor is defined as the ratio between the observed counts and the counts expected by chance. The most statistically significant term within a cluster was selected to represent the cluster.

### Statistical analysis

Statistical analyses were conducted using the R software (version 4.1.2) or GraphPad Prism (version 9.0.0). The 28-day survival curves of the high- and low-risk groups were drawn according to the Kaplan–Meier estimate, and the survival differences were compared using the log-rank test. Univariate and multivariate Cox regression analyses were performed to investigate whether this gene signature was an independent determinant of 28-day survival. Receiver operating characteristic (ROC) analysis was used to evaluate the sensitivity and specificity of the survival prediction based on the risk score, indexes of other gene signatures, and combined models. The area under the ROC curve (AUC) was used to measure the accuracy of the prediction test and the DeLong method was used to assess differences between the ROC curves. The Wilcoxon rank-sum test was used to compare risk scores between two groups, while the Kruskal–Wallis test was used for comparison among more than two groups. Spearman’s correlation analysis was applied to assess the association between the risk score and immune cell abundance. Fisher's exact test was carried out for comparison of 28-day mortality or other clinical features between the high- and low-risk groups. Hypothesis testing with a two-tailed *P*-value < 0.05 was considered statistically significant.

## Results

### Development and validation of a prognostic HLMRG signature

**Fig. 1:**
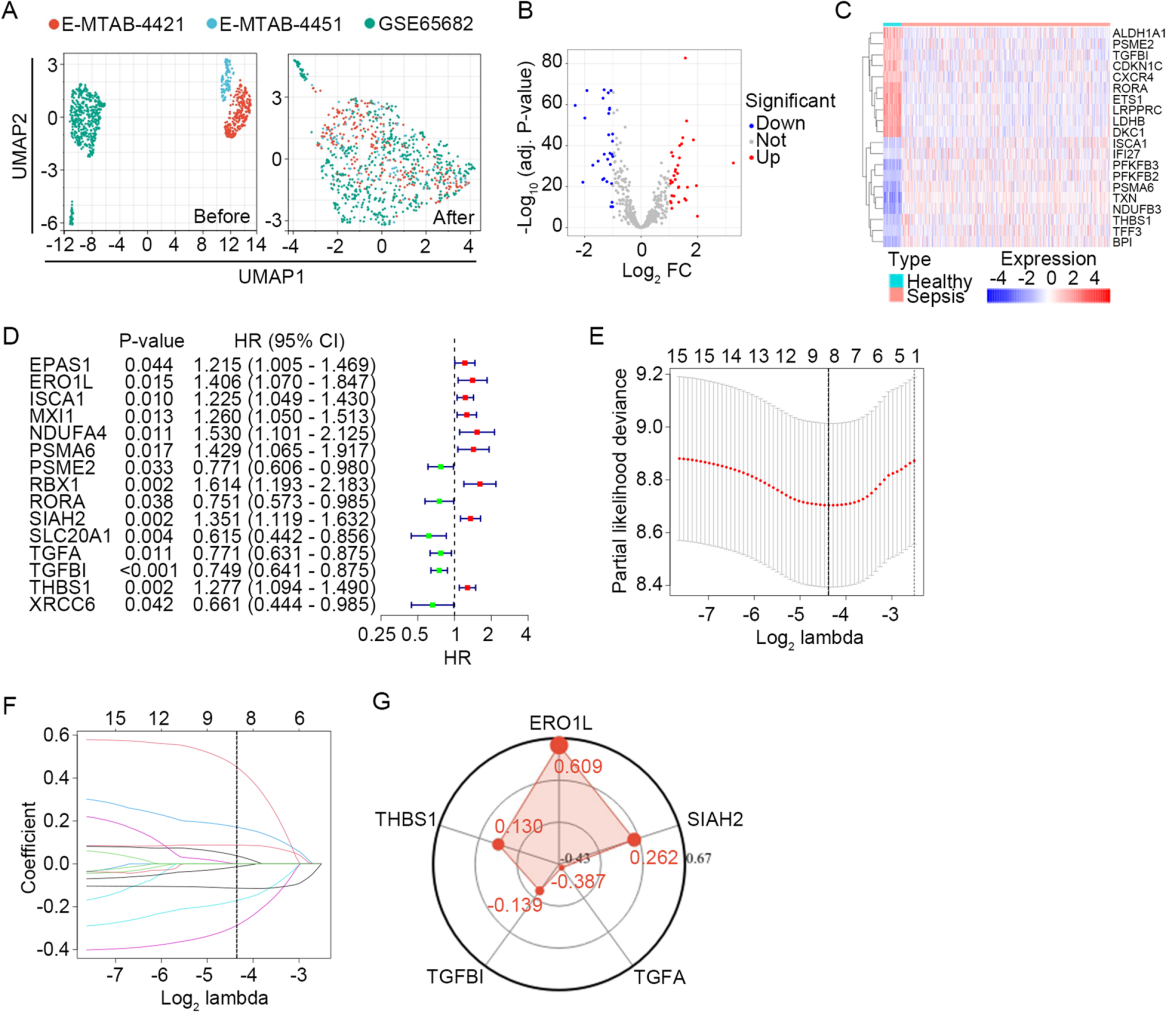
Construction of a prognostic HLMRG signature. **A** UMAP algorithm showing sample clusters before and after batch normalization. **B** Volcano plot showing differentially expressed HLMRGs between sepsis patients (*n* = 479) and healthy controls (*n* = 42) in the discovery set. The screening criteria were set as adjusted *P* < 0.05 and | log_2_ (fold change) |≥ 1. **C** Expression profile of the top 10 dysregulated HLMRGs. **D** Univariate Cox regression analysis to screen HLMRGs related to 28-day survival of sepsis patients in the discovery set (*n* = 479). Squares represent the HR of death and open-ended horizontal lines represent the 95% CI. All P-values were calculated using Cox proportional hazards analysis. **E** Ten-fold cross-validation for tuning parameter selection in the LASSO estimation. The partial likelihood deviance corresponding to each lambda value is shown as mean ± SD. The dotted vertical line (left) indicates the optimal value by minimum criteria. **F** LASSO coefficient profile of individual genes included in the estimation. **G** Distribution of the multivariate Cox regression coefficients of the HLMRG signature

First, the three datasets included in this study (GSE65682, E-MTAB-4421, and E-MTAB-4451) were merged to eliminate batch effects (Fig. 1A). Next, we performed differential expression analysis in the GSE65682 dataset, which was designated as the discovery set. A set of 61 differentially expressed HLMRGs between healthy controls (*n* = 42) and sepsis patients (*n* = 479) were screened, with 29 HLMRGs downregulated and 32 HLMRGs upregulated (Fig. 1B). The expression profile of the top 10 differentially expressed HLMRGs is shown in Fig. 1C. Univariate Cox regression analysis was employed to further assess the 61 HLMRGs in the discovery set. A set of 15 HLMRGs was significantly associated with the 28-day survival of sepsis patients (Fig. 1D). Seven of these 15 HLMRGs were excluded by LASSO estimation because of multicollinearity (Fig. 1E). Finally, a prognostic HLMRGs signature consisting of five genes (*ERO1L*, *SIAH2*, *TGFA*, *TGFBI*, and *THBS1*) was identified by multivariate Cox regression analysis. Each patient’s risk score was determined according to the expression levels of the five selected genes and their associated coefficients in the multivariate Cox model. For patients with sepsis, a higher risk score indicated poorer 28-day survival. Among the five HLMRGs, *ERO1L*, *SIAH2*, and *THBS1* showed positive coefficients, whereas *TGFA* and *TGFBI* showed negative coefficients (Fig. 1F). Higher expression levels of genes with positive weighting coefficients indicated worse outcomes, whereas higher expression levels of genes with negative weighting coefficients suggested better outcomes.

**Fig. 2:**
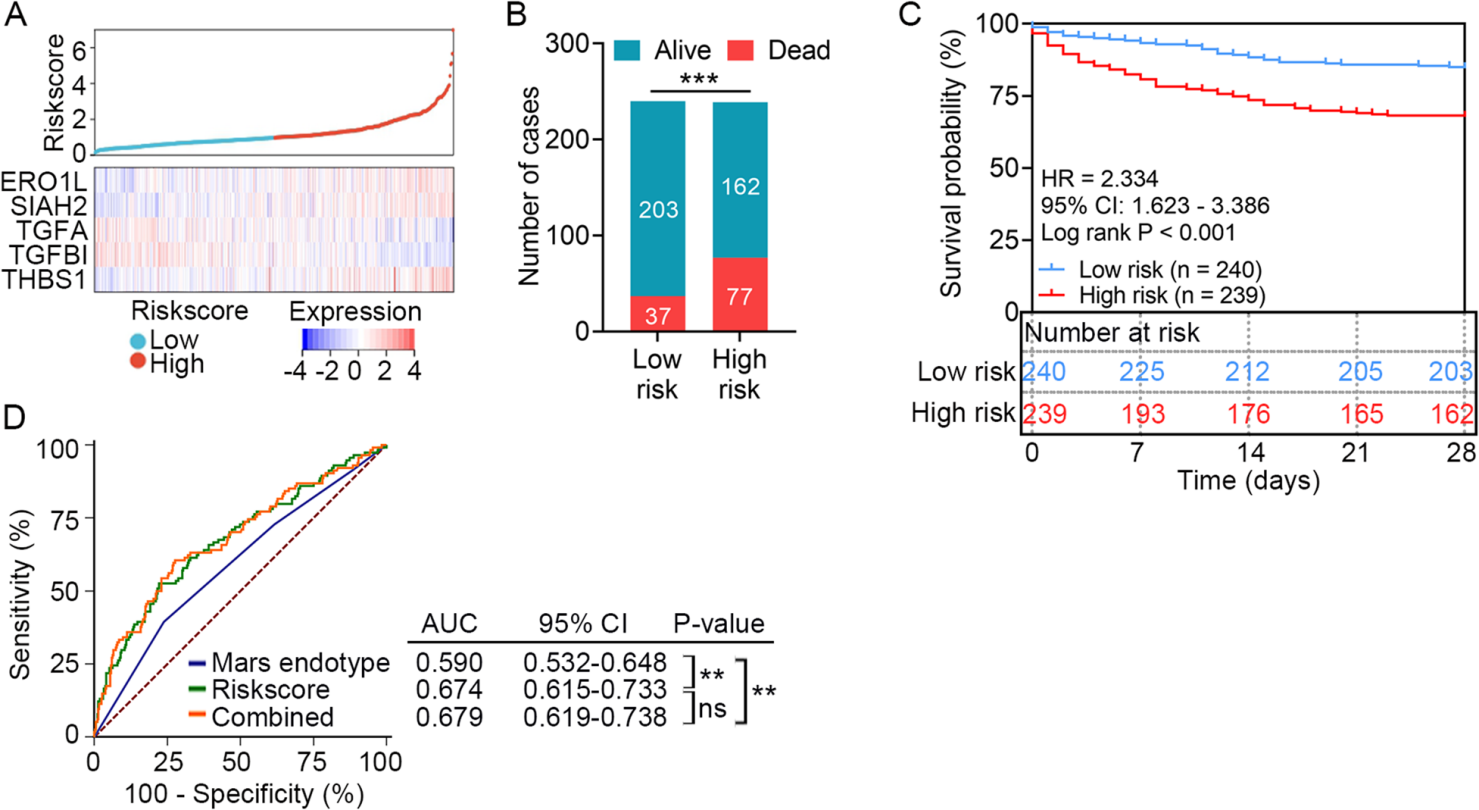
Survival analysis of the HLMRG signature in the discovery set (*n* = 479). **A** Distribution of risk scores derived from the HLMRG signature, and expression profile of the five genes that comprised the HLMRG signature in the discovery set. **B** Distribution of 28-day survival status in the high-risk (*n* = 239) and low-risk (*n* = 240) groups. Sepsis patients were classified into different risk groups using the median risk score as the cutoff. **C** Kaplan–Meier estimate of the 28-day survival according to the HLMRG signature. The difference between the two curves was determined by the two-side log-rank test. **D** ROC analysis of the sensitivity and specificity of 28-day survival prediction by the HLMRG signature risk score, Mars endotype, and combination of the two factors. *P*-values were obtained from the pairwise comparisons of the AUCs using the Delong method. **P < 0.01, ***P < 0.001, *ns* no significance

We profiled the distribution of risk scores and mRNA expression of the five genes consisting of the HLMRG signature in the discovery set. The results showed that the expression levels of genes with positive coefficients were higher in high-risk patients (Fig. 2A). Patients were divided into high-risk (*n* = 239) and low-risk (*n* = 240) groups according to the median risk score. The 28-day survival status of sepsis patients in the high- and low-risk groups is shown in Fig. 2B. A significantly higher mortality rate was found in the high-risk group compare to the low-risk group (32.22% vs. 15.42%, *P* < 0.001). Kaplan–Meier analysis revealed that sepsis patients in the high-risk group demonstrated worse outcomes than those in the low-risk group (HR = 2.334, 95% CI 1.623–3.386, *P* < 0.001; Fig. 2C). In GSE65682, four molecular classifications designated as the molecular diagnosis and risk stratification of sepsis (Mars) endotypes were identified based on transcriptomic profile of PBMCs from sepsis patients [22]. The Mars endotype has been proven as a sufficient predictor of 28-day mortality for sepsis patients, and patients with Mars1 endotype tended to have the worst outcome. As shown in Fig. 2D, the AUC of the HLMRG signature was significantly higher than that of the Mars endotype (0.674 vs. 0.590, *P* = 0.007). Moreover, the AUC of the HLMRG signature combined with the Mars endotype was significantly higher than that of the Mars endotype alone (0.679 vs. 0.590, *P* = 0.003).

**Fig. 3:**
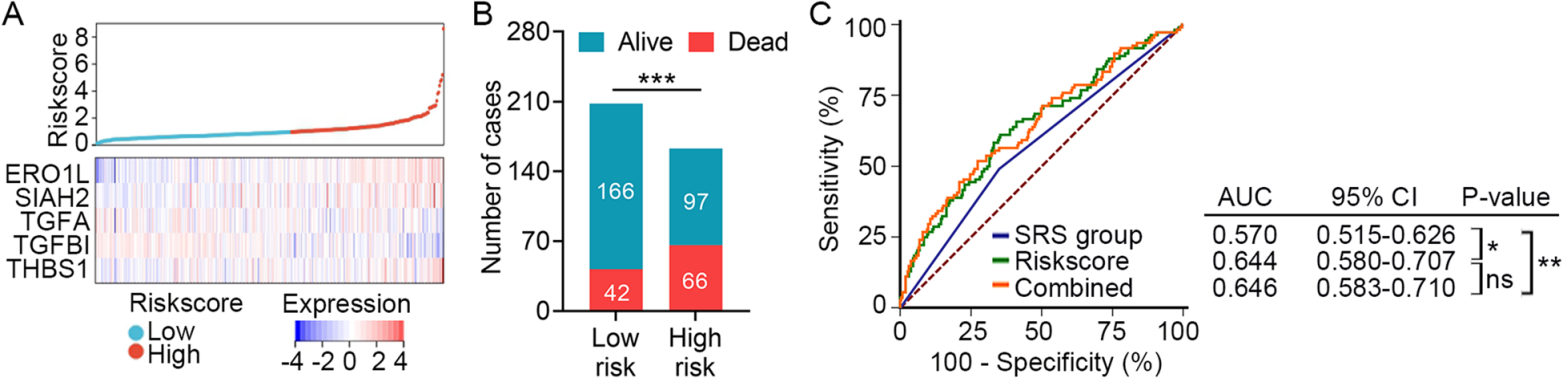
Survival analysis of the HLMRG signature in the validation set (*n* = 371). **A** Distribution of risk scores derived from the HLMRG signature, and expression profile of the five genes that comprised the HLMRG signature in the validation set. **B** Distribution of 28-day survival status in the high-risk (*n* = 163) and low-risk (*n* = 208) groups. Sepsis patients were classified into different risk groups based on the same cutoff used in the discovery set. **C** ROC analysis of the sensitivity and specificity of 28-day survival prediction by the HLMRG signature risk score, SRS group, and combination of the two factors. *P-*values were obtained from the pairwise comparisons of the AUCs using the Delong method. *P < 0.05, **P < 0.01, ***P < 0.001, *ns* no significance

The efficacy of the HLMRG signature for predicting 28-day mortality of sepsis patients was further confirmed in a validation set (E-MTAB-4421 plus E-MTAB-4451). The cut-off value for patient classification was also set as the median risk score of the discovery set. Consistent with the findings described above, higher expression levels of *ERO1L*, *SIAH2*, and *THBS1*, whereas lower expression levels of *TGFA* and *TGFBI* were observed in patients with higher risk scores (Fig. 3A). Furthermore, a higher mortality rate was observed in the high-risk group (*n* = 163) than that in the low-risk group (*n* = 208) (40.49% vs. 20.19%, *P* < 0.001; Fig. 3B). In E-MTAB-4421 and E-MTAB-4451, two distinct sepsis response signature groups (SRS1 and SRS2) were defined based on transcriptomic analysis of PBMCs from sepsis patients [23]. Patients classified in SRS1 had higher short-term (14 day and 28 day) and long-term (6 month) mortality was than those belonged to SRS2. As shown in Fig. 3C, the AUC of the HLMRG signature was significantly higher than that of the SRS group (0.644 vs. 0.570, *P* = 0.041). Furthermore, the AUC of the HLMRG signature combined with SRS group was significantly higher than that of SRS group alone (0.646 vs. 0.570, *P* = 0.004). These results demonstrated that the identified HLMRG signature is a reliable classifier that can discriminate patients with sepsis into risky groups with significantly disparate 28-day survival.

### Prognostic value of the HLMRG signature

**Fig. 4:**
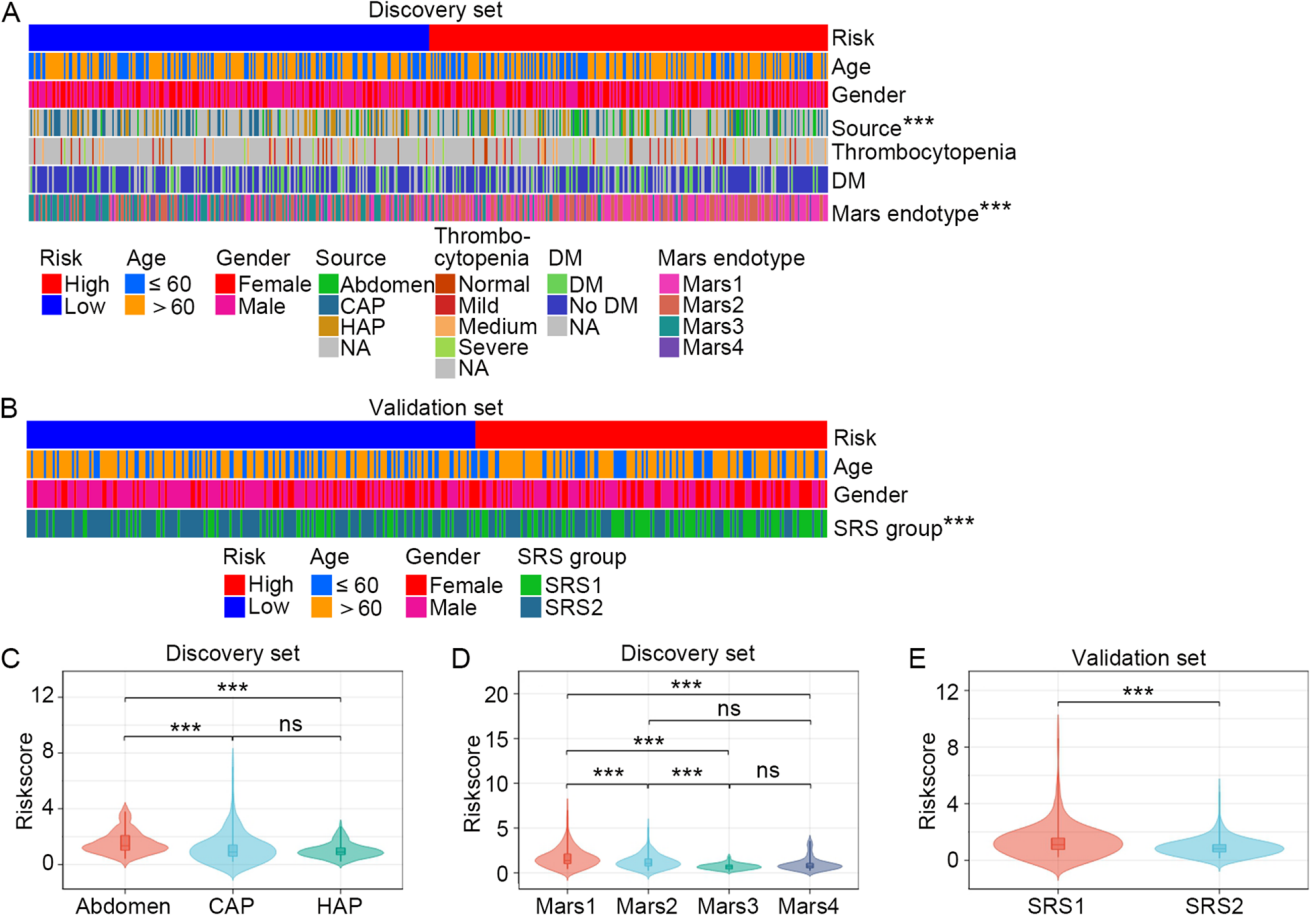
Association between the HLMRG signature and clinical features of sepsis patients. **A** Heatmap showing correlation of the HLMRG signature with age, gender, source of infection, thrombocytopenia, diabetes mellitus and Mars endotype in the discovery set (*n* = 479). NA, not available. **B** Heatmap showing correlation of the HLMRG signature with age, gender and SRS group in the validation set (*n* = 371). Comparisons of the risk score in sepsis patients with disperse source of infection (**C**), Mars endotype (**D**), and SRS group (**E**). The Wilcoxon rank-sum test was used to compare risk scores between two groups, while the Kruskal–Wallis test was applied for comparisons among more than two groups. ***P < 0.001, *ns* no significance

First, the correlation between clinical features and the HLMRG signature was investigated using Fisher's exact test. In the discovery set, the HLMRG signature significantly correlated with Mars endotype and the source of infection, but not with age, gender, history of diabetes mellitus, or thrombocytopenia (Fig. 4A). In the validation set, the HLMRG signature significantly correlated with the SRS group, but not with age or gender (Fig. 4B). We also compared the risk scores in patients with the dispersed Mars endotype, source of infection, and SRS group. These results showed that significantly higher risk scores were manifested in patients with an abdominal infection source (Fig. 4C), Mars 1 endotype (Fig. 4D), or the SRS1 group (Fig. 4E).

**Fig. 5:**
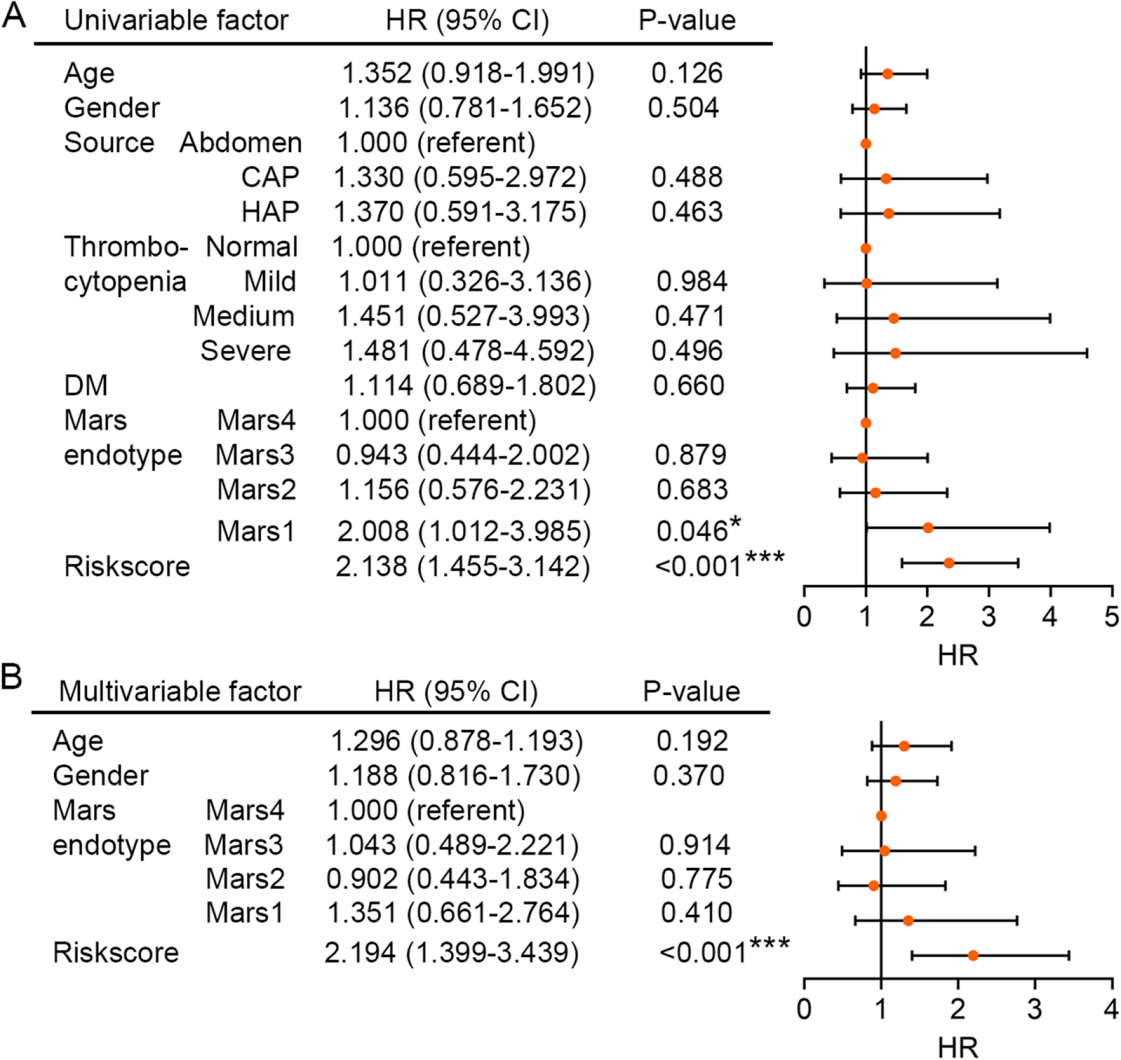
The HLMRG signature is an independent prognostic factor of sepsis patients. **A** Univariate Cox regression analysis performed on sepsis patients in the discovery set (*n* = 479). Orange solid dots represent the HR of death and open-ended horizontal lines represent the 95% CIs. All *P*-values were calculated using Cox proportional hazards analysis. **B** Multivariate Cox regression analysis that contained age, gender, Mars endotype, and HLMRG signature as covariates. *P < 0.05, ***P < 0.001

Next, univariate Cox regression analysis was employed to the discovery set. The results showed that the Mars endotype (Mars1, HR = 2.008, 95% CI 1.012–3.985, *P* = 0.046) and HLMRG signature (HR = 2.347, 95% CI 1.585–3.474, *P* < 0.001) both significantly correlated with the 28-day survival of sepsis patients (Fig. 5A). Finally, multivariate Cox regression analysis with age, gender, Mars endotype, and HLMRG signature as covariates revealed that the HLMRG signature showed independence in predicting the 28-day survival of sepsis patients (HR = 2.194, 95% CI 1.399–3.439, *P* < 0.001; Fig. 5B). These results suggested the identified HLMRG signature as an independent prognostic factor for sepsis patients.

### Association between the HLMRG signature and immune cell subtypes

**Fig. 6:**
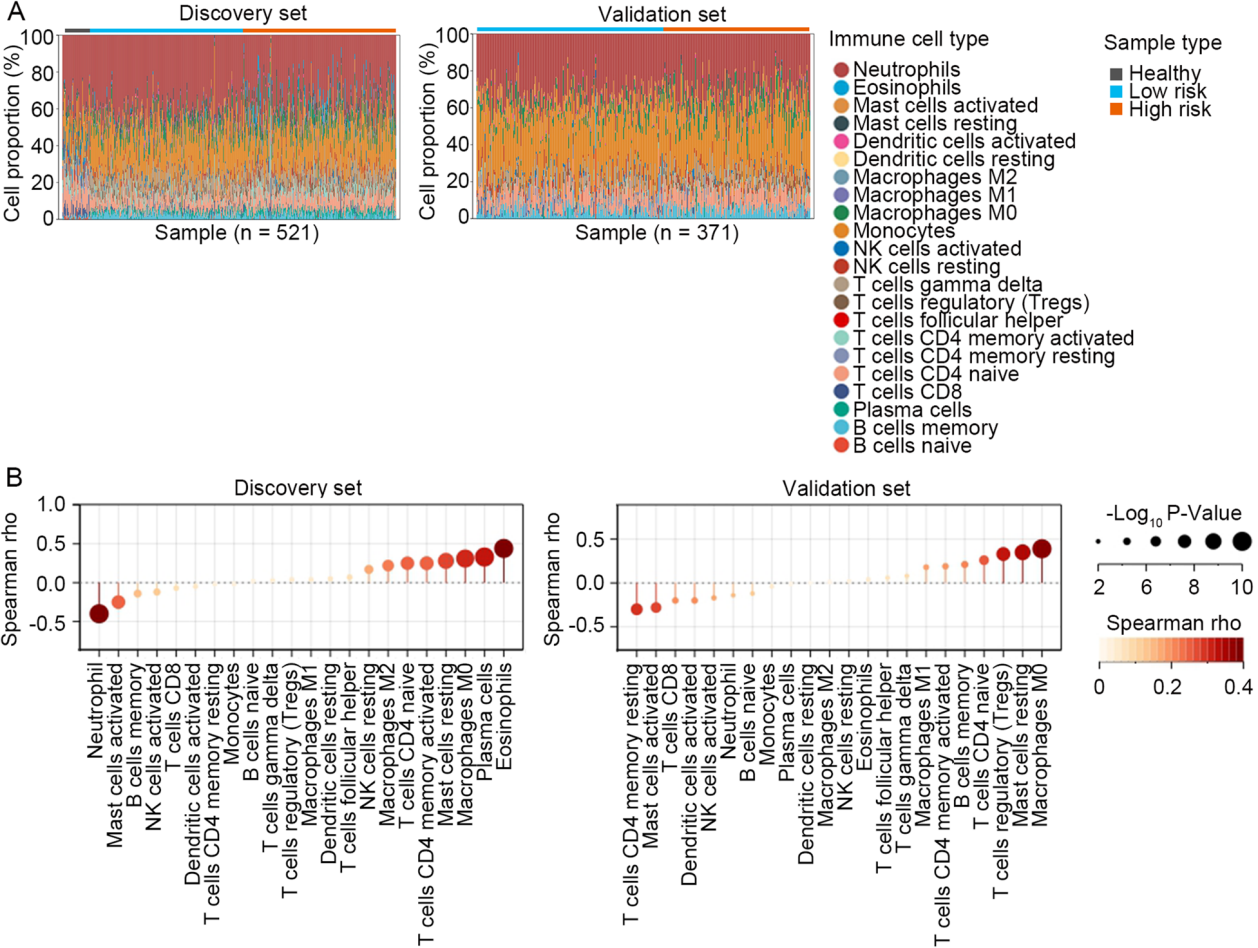
Association between the HLMRG signature and immune cell subtypes. **A** Immune cell fractions of sepsis patients in the discovery set (*n* = 479) and validation set (*n* = 371) calculated by the CIBERSORT method. **B** The correlation between the HLMRG risk score and immune cell fractions analyzed by Spearman’s correlation analysis

Lactate is recognized as a metabolic player involving in immune cell fate, and a key player in determining immune cell fate and regulating immune cell function [34]. Therefore, we investigated the correlation between the HLMRG signature and immune cell abundance in sepsis. First, the immune cell proportions in PBMCs of sepsis patients were calculated using the CIBERSORT method. Based on this algorithm, we found that patients with sepsis displayed heterogeneous enrichment of immune cell populations (Fig. 6A). Next, the correlation between the HLMRG signature and immune cell fraction was evaluated using Spearman’s correlation analysis. In the discovery set, the results suggested that the proportions of eosinophils, plasma cells, and M0 macrophages were positively associated with the risk score, whereas the proportion of neutrophils negatively correlated with the risk score (|Spearman rho|> 0.30, *P* < 0.001; Fig. 6B, left panel). In the validation set, the results revealed that the abundance of M0 macrophages, resting mast cells, and regulatory T cells positively correlated with the risk score, whereas the abundance of resting memory CD4^+^ T cells negatively correlated with the risk score (|Spearman rho|> 0.30, *P* < 0.001; Fig. 6B, right panel). The above results uncovered a consistent positive correlation between the HLMRG signature and the abundance of M0 macrophages in both datasets, indicating the involvement of hypoxia and lactate metabolism in regulating macrophage function, as well as the role of macrophages in determining the prognosis of sepsis patients.

### Assessment of biological pathways related to the HLMRG signature

**Fig. 7:**
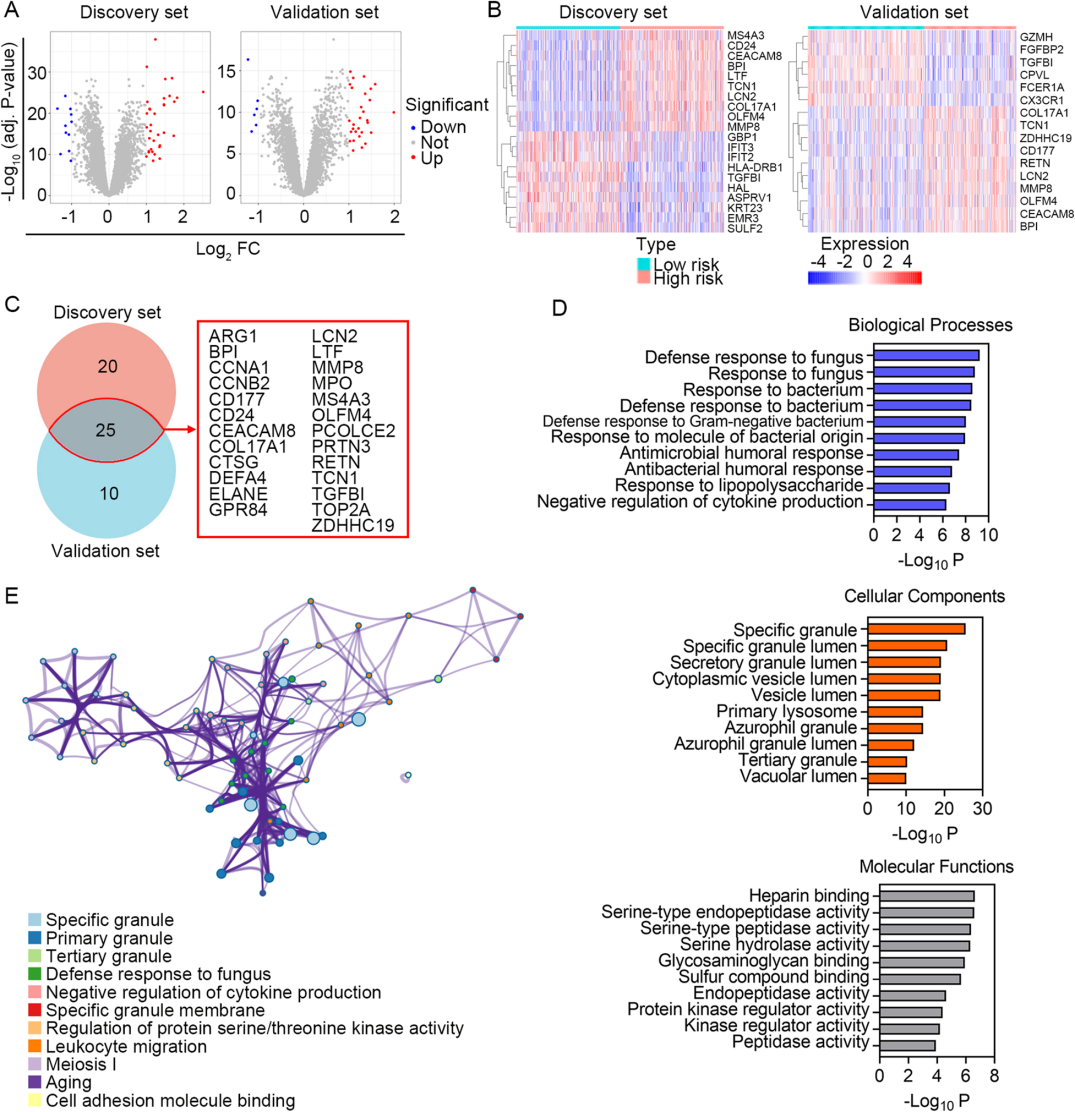
Assessment of biological pathways related to the HLMRG signature. **A** Volcano plots showing differentially expressed genes between high and low risk in the discovery and validation sets. The screening criteria were set as adjusted *P* < 0.05 and | log_2_ (fold change) |≥ 1. **B** Expression profile of top 10 DEGs between high and low risk groups in the discovery and validation sets. **C** Venn diagram of intersected DEGs screened from the discovery and validation sets. **D** GO analysis of the identified DEGs. Top 10 significantly enriched terms related to Biological Processes, Cellular Components and Molecular Functions were shown. **E** Network plot showing relationships between the enriched terms. Nodes represent enriched gene sets that are grouped and annotated by their similarity according to related gene sets. Node size is proportional to the total number of genes within each gene set. Proportion of shared genes between gene sets is represented as the thickness of the connecting line between nodes

To investigate the potential biological pathways associated with the HLMRG signature, we performed GO analysis based on DEGs between the high- and low-risk groups. 45 and 35 DEGs were screened out in the discovery and validation datasets, respectively (Fig. 7A). The expression profile of the top 10 DEGs between high and low risk groups in the discovery and validation sets are shown in Fig. 7B. Intersection analysis yielded 25 DEGs that were coincidently dysregulated in both of the datasets (Fig. 7C). GO analysis was performed based on these 25 DEGs using Metascape, a free online tool for gene annotation and functional enrichment. Genes enriched for biological processes were mainly involved in the response to fungus/bacteria, antimicrobial humoral response, and regulation of cytokine production (Fig. 7D, upper panel). Genes enriched for cellular components were principally associated with the specific/secretory/tertiary granule/vesicle lumen and primary lysosomes (Fig. 7D, middle panel). Genes enriched for molecular functions were predominantly related to heparin binding, peptidase/endopeptidase activity, and kinase regulator activity (Fig. 7D, lower panel). As shown in Fig. 7E, the enriched terms were classified into 11 clusters according to their membership similarities. The full list of the terms associated with the HLMRG signature is summarized in Additional file 3: Table S3.

## Discussion

The recent sepsis-3 guidelines have proposed that sepsis should be defined as a life-threatening organ dysfunction caused by a dysregulated host response to infection [1]. There is also a recommendation that the definition of septic shock should include the persistence of a serum lactate level more than 2 mmol/L despite adequate fluid resuscitation as a new criterion [1]. This recommendation is proposed on the basis of the clinical observation that lactate concentrations are strongly correlated with severity, morbidity, and mortality of sepsis. However, the molecular consequences of hypoxia and the pathophysiological processes of lactate metabolism and clearance remain poorly understood, and further research is required. Herein, we extensively assessed the expression of a set of dysregulated HLMRGs in sepsis, based on which a prognostic HLMRG signature was developed and validated in a large number of participants (total *n* = 850). The HLMRG signature was established by combining the Cox regression model with LASSO regularization for parameter shrinkage, and a prognostic model consisting of only five genes (*ERO1L*, *SIAH2*, *TGFA*, *TGFBI*, and *THBS1*) was obtained. The minimal number of gene members indicates their clinical practicability and economic advisability. Clear survival differences in 28-day mortality between patients in the high- and low-risk groups classified by the HLMRG signature were observed in both the discovery set (*n* = 479) and the validation set (*n* = 371). Four molecular endotypes associated with 28-day mortality of sepsis were identified by previous study based on transcriptomic profile of PBMCs from sepsis patients; patients with a Mars1 endotype had the worst outcome [22]. In the current study, the HLMRG signature significantly correlated with the Mars endotype. This correlation suggested Mars endotype and HLMRG were both potential prognostic factors for sepsis, which was verified by the univariate Cox regression analysis. However, the multivariate Cox regression analysis with age, gender, Mars endotype, and HLMRG signature as covariates revealed that only the HLMRG signature could be an independent determinant for predicting the 28-day mortality of sepsis patients. These results suggested the prominent reliability of the identified HLMRG signature for predicting sepsis outcomes. We further performed ROC analysis to evaluate the sensitivity and specificity of the survival prediction, and the AUC of the HLMRG signature was significantly higher than that of the Mars endotype, but combination of the HLMRG signature and Mars endotype could not further increased the predicting capacity. We inferred the reason was that these two predictive signatures were both derived from transcriptomic analysis, and the inclusion of meaningful clinical characteristics may greatly improve the predicting efficacy.

**Fig. 8:**
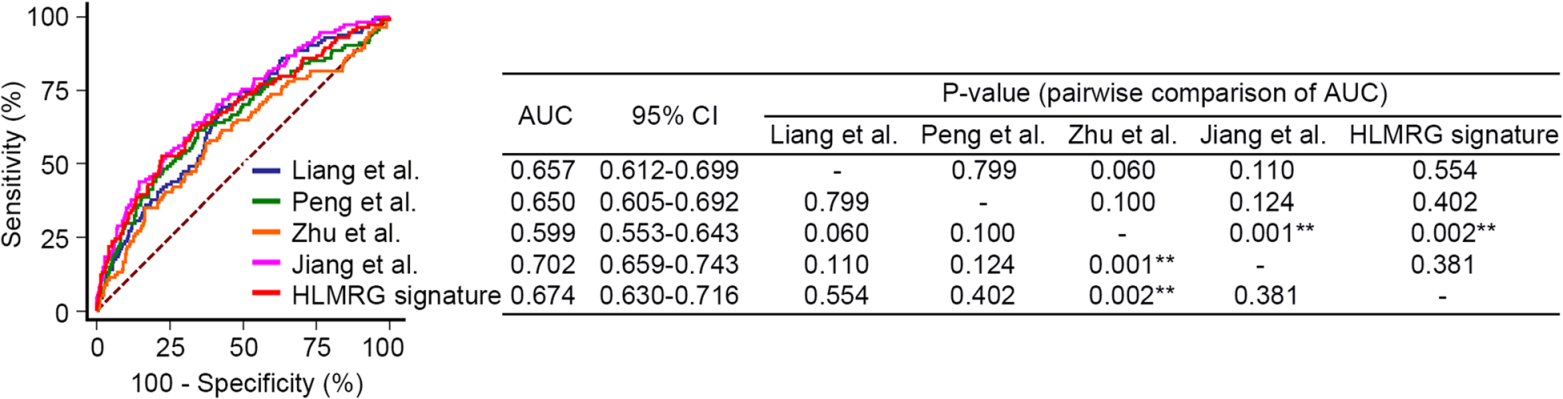
Comparison of the AUC of the HLMRG signature with those of the other groups. **P < 0.01

Several investigators have successfully subgroup patients with sepsis based on genetic signatures derived from transcriptomic profiling of PBMCs. Liang et al. [35] developed and validated a novel prognostic predictive risk score for sepsis based on six pyroptosis-related genes (*GZMB*, *CHMP7*, *NLRP1*, *MYD88*, *ELANE*, and *AIM*). Four out of the six genes (*GZMB*, *CHMP7*, *NLRP1*, and *AIM2*) also have potential diagnostic value in sepsis diagnosis [35]. Peng et al. [27] established a prognostic immune-related gene signature comprising three gene members (*LTB4R*, *HLA-DMB* and *IL4R*). This prognostic signature demonstrated good predictive performance for 28-day mortality in the internal and external validation datasets [27]. Zhu et al. [36] identified a ferroptosis-related prognostic signature (*TLR4*, *WIPI1*, and *GABARAPL2*), and Jiang et al. [37] developed a signature consisting of nine inflammatory response-related genes (*CCL22*, *CX3CL1*, *CXCR6*, *FFAR2*, *FPR1*, *HBEGF*, *ITGA5*, *RGS16*, and *SELL*) to predict prognosis. The AUC of the HLMRG signature was comparable to the AUCs of the signatures identified by the groups aforementioned, except that it was significantly higher than that of the signature identified by Zhu and his colleagues (0.674 vs. 0.599, *P* = 0.002; Fig. 8).

Lactate is traditionally recognized as a byproduct derived from glucose metabolism. More recently, there is growing evidence indicates that role of lactate is a key player in the regulation of various biological and pathological processes. Infection, inflammation, hypoxia, and tumors are found to promote lactate production [38]. A plethora of studies have highlighted that lactate play a role in regulating a wide range of immune cells involved in maintaining host immune homeostasis [38]. In sepsis, aerobic glycolytic metabolism fundamentally participated in activation of immune cell, and the lactate produced by aerobic glycolysis plays an immunosuppressive role [7]. The biological effects of lactate on innate myeloid cells have been extensively studied. In monocytes and macrophages, lactic acid suppresses an arrangement of lipopolysaccharide-induced (LPS)-induced cytokines and chemokine mediators [39–41]. Lactate itself also acts as a suppressor of inflammasome assembly, LPS-stimulated cytokine secretion, and migration of macrophages and monocytes [42–44]. In the present study, we inferred from the transcriptomic data a consistent positive correlation between the HLMRG signature and the abundance of M0 macrophages in the discovery and validation sets, indicating the involvement of hypoxia and lactate metabolism in regulating macrophage function, as well as the role of macrophages in determining the prognosis of sepsis patients. Finally, DEGs between the high- and low-risk groups were screened out for GO analysis, and the results suggested that these DEGs were significantly associated with pathway clusters of cell adhesion molecular binding, cytokine production, protein serine or threonine kinase activity, and leukocyte migration, providing inspiration for us to further discover the underlying biological mechanisms of the HLMRG signature.

The current study has few limitations. First, this was a retrospective study, and transcriptomic data were retrieved from primarily available researches; we were unable to control for infection source, demographics, or patient severity. Second, the HLMRG signature was established merely on the basis of transcriptomic data. The AUCs of the HLMRG signature were 0.674 and 0.644 for the discovery and validation sets, respectively, although the prediction performance was better than that of existing molecular biomarkers such as the Mars endotype and the SRS group. The AUCs are relatively low for a clinically useful biomarker; therefore, the clinical characteristics of patients with sepsis or other system-based omics data should also be added to the model to increase its predictive power. Third, despite validation in an independent cohort, we did not evaluate the HLMRG signature in any clinical study conducted at our medical center. A prospective analysis should be designed for translational purposes of the HLMRG signature. Finally, there existed few experimental data regarding the functional roles and underlying molecular mechanisms of the genes consisting of the HLMRG signature. As such, detailed experimental validation and exploration should be designed to gain insights into the roles and mechanisms of the genes consisting of the HLMRG signature.

## Conclusions

The current study identified a group of HLMRGs dysregulated in sepsis patients. Using these genes, a prognostic model was developed and tested, which accurately predicts the likelihood of 28-day mortality in sepsis patients. This gene expression model is reflective of the underlying biological response of sepsis and the immune state of sepsis patients. Prospective clinical investigations and targeted studies of individual genes and relevant pathways are required to confirm and extend the findings presented here.

### Supplementary Information

**Additional file 1: Table S1.** Baseline characteristics of sepsis patients included in this study.

**Additional file 2: Table S2.** A list of 764 HLMRGs retrieved from the Molecular Signatures Database.

**Additional file 3: Table S3.** The full list of enriched terms associated with the HLMRG signature.

## Data Availability

Clinical information and gene expression profiles included in this study can be accessed in GEO (http://www.ncbi.nlm.nih.gov/geo/) and ArrayExpress (https://www.ebi.ac.uk/arrayexpress/), which are publicly available databases.

## Abbreviations

ROC: Receiver operating characteristic
HLMRGs: Hypoxia- and lactate metabolism-related genes
AUC: Area under the ROC curve
GO: Gene ontology
DEGs: Differentially expressed genes
LASSO: Least absolute shrinkage and selection operator
SRS: Sepsis response signature
PBMCs: Peripheral blood mononuclear cells
Mars: Molecular diagnosis and risk stratification of sepsis
