# Supplementary material for: A hypoxia- and lactate metabolism-related gene signature to predict prognosis of sepsis: discovery and validation in independent cohorts
Source: Eur J Med Res. 2023 Sep 4;28:320. doi: 10.1186/s40001-023-01307-z (PMC10476321; doi:10.1186/s40001-023-01307-z)
Supplement: Supplementary file 1 — Additional file 1: Table S1. Baseline characteristics of sepsis patients included in this study. [file 40001_2023_1307_MOESM1_ESM.docx]

**Table S1.** Baseline characteristics of sepsis patients included in this study.

| **GSE65682 (n = 479)** |  |
| --- | --- |
| Age, years | 60.95 (14.79) |
| Gender |  |
| Male | 272 (56.78%) |
| Female | 207 (43.22%) |
| DM |  |
| No | 301 (62.84%) |
| Yes | 89 (18.58%) |
| NA | 89 (18.58%) |
| Source of infection |  |
| CAP | 106 (22.13%) |
| HAP | 77 (16.08%) |
| Abdomen | 48 (10.02%) |
| NA | 248 (51.77%) |
| Thrombocytopenia |  |
| Normal | 24 (5.01%) |
| Mild | 24 (5.01%) |
| Medium | 30 (6.26%) |
| Severe | 17 (3.55%) |
| NA | 384 (80.17%) |
| Mars endotype |  |
| Mars1 | 132 (27.56%) |
| Mars2 | 176 (36.74%) |
| Mars3 | 118 (24.63%) |
| Mars4 | 53 (11.07%) |
| 28-day mortality event |  |
| Alive | 365 (76.20%) |
| Dead | 114 (23.80%) |
| Follow-up time, days | 23.19 (9.20) |
| **E-MTAB-4421 & E-MTAB-4451 (n = 371)** |  |
| Age, years | 63.87 (15.96) |
| Gender |  |
| Male | 224 (60.38%) |
| Female | 147 (39.62%) |
| SRS group |  |
| SRS1 | 145 (39.08%) |
| SRS2 | 226 (60.92%) |
| 28-day mortality event |  |
| Alive | 263 (70.89%) |
| Dead | 108 (29.11%) |

Data are n (%) or mean (SD) unless otherwise specified. DM, Diabetes mellitus; Mars, the Molecular Diagnosis and Risk Stratification of Sepsis; SRS, sepsis response signature; NA, not available.
